# Predictors of in-hospital mortality after posterior fossa decompression for cerebellar stroke: an exploratory cohort study

**DOI:** 10.1016/j.bas.2026.107721

**Published:** 2026-11-01

**Authors:** Sergio Alexander Calero Martinez, Daniel Sarmiento, Ricardo Borda, Christian Senft, Falko Schwarz, Nazife Dinc, Nazeer Aboud

**Affiliations:** a Department of Neurosurgery, Jena University Hospital, Friedrich-Schiller-University Jena, Jena, Germany; b Department of Statistics, El Bosque University, Bogota, Colombia; c Instituto de Neurociencias, Faculty of Medicine, El Bosque University, Bogotá, Colombia

**Keywords:** Cerebellar hemorrhage, Cerebellar infarction, Posterior fossa decompression, Cerebellar stroke, In-hospital mortality, Fourth ventricle compression

## Abstract

**Background:**

Surgical intervention for cerebellar insult remains a critical yet high-risk endeavor. This study aimed to explore associations between clinical and volumetric variables and in-hospital mortality in patients undergoing posterior fossa decompression in a hypothesis-generating cohort.

**Methods:**

We performed a retrospective analysis of 23 patients treated surgically for cerebellar hemorrhage or infarction. A LASSO (Least Absolute Shrinkage and Selection Operator) logistic regression was employed to screen and rank candidate variables associated with death. Variables analyzed included demographics, type of stroke, lesion location (unilateral vs. bilateral/midline), 4th ventricle compression, pre-surgical Glasgow Coma Scale (GCS), and quantitative cerebellar volumes.

**Results:**

In univariate analyses, type of stroke was associated with survival in this cohort (p = 0.014), with deaths observed only among patients with hemorrhage. Bilateral or midline involvement was also associated with mortality (p = 0.012). In contrast, pre-surgical GCS and volumetric measures of the lesion and posterior fossa were not significantly associated with mortality. Exploratory LASSO analysis showed positive mortality coefficients for hemorrhage (OR 4.4) and bilateral or midline-crossing lesions (OR 3.6).

**Conclusions:**

In this exploratory cohort, mortality was more frequently observed in patients with hemorrhagic pathology and bilateral/midline-crossing involvement. These findings are hypothesis-generating and require confirmation in larger cohorts.

## Highlights

• Hemorrhagic cerebellar stroke was associated with higher in-hospital mortality compared with cerebellar infarction.
• Bilateral or midline-crossing cerebellar lesions were associated with markedly higher mortality.
• Fourth-ventricle compression emerged as a key radiological marker of critical posterior fossa compromise.
• Quantitative volumetric parameters showed no association with early postoperative mortality.
• Simple anatomical imaging markers may outperform complex volumetric analyses for risk stratification.

## 1 Introduction

Cerebellar insults resulting from ischemic or hemorrhagic stroke, traumatic brain injury, or other acute pathological processes represent a distinct clinical entity associated with substantial morbidity and mortality. Reported long-term case fatality rates after cerebellar hemorrhage vary widely, ranging from 0% to 50%, reflecting heterogeneity in patient characteristics, lesion severity, and therapeutic strategies (Senff et al., 2024). Despite advances in neurocritical care and neurosurgical techniques, outcomes remain highly variable, underscoring the need for improved risk stratification.

When clinical deterioration, mass effect, hydrocephalus, or brainstem compression occurs, surgical intervention is often required. Procedures such as posterior fossa decompression, hematoma evacuation, or cerebrospinal fluid (CSF) diversion aim to reduce intracranial pressure and prevent secondary injury. However, these interventions are themselves associated with significant perioperative risk and non-negligible mortality (Al Safatli et al., 2017; Arnone et al., 2017). Prior studies have identified several clinical and radiographic predictors of outcome, including level of consciousness, hematoma size, ventricular compression, and hydrocephalus, yet consistent predictors of mortality remain elusive (Dahdaleh et al., 2012).

The cerebellum's complex anatomy and functional compartmentalization further complicate surgical decision-making. Lesion location, extent of midline involvement, and distortion of posterior fossa structures may critically influence both surgical strategy and prognosis, highlighting the importance of individualized treatment approaches (Tsitsopoulos et al., 2011). Although previous studies have investigated mortality-related factors in cerebellar hemorrhage and ischemic stroke, differences in patient selection, surgical management, and outcome definitions limit their direct applicability to patients undergoing posterior fossa decompression (Al Safatli et al., 2017; Arnone et al., 2017; Won et al., 2023). Moreover, evidence specifically addressing the association between quantitative posterior fossa measurements and in-hospital mortality in surgically treated cohorts remains limited (Won et al., 2023).

Overall, existing research provides incomplete clinical and paraclinical data regarding mortality after cerebellar insults, particularly with respect to quantitative neuroimaging parameters. In particular, the prognostic value of volumetric measurements of the posterior fossa and cerebellum has not been systematically explored in surgical cohorts. The rationale for assessing lesion and compartment volumes was that an identical lesion volume may have different space-occupying implications depending on the available posterior fossa space.

Therefore, the present study aims to evaluate the association between clinical, anatomical, and volumetric factors and in-hospital mortality in adult patients undergoing surgical treatment after cerebellar insult. By integrating demographic variables, clinical presentation, and detailed neuroimaging measurements, this study seeks to identify candidate signals associated with death and to inform variable selection for future, adequately powered prognostic studies.

## 2 Methods

### 2.1 Study design and population

We conducted a retrospective, single-center cohort study of 23 consecutive adults who underwent suboccipital decompression for cerebellar infarction, hemorrhage, or hemorrhagic transformation between January 2015 and August 2024 at the Department of Neurosurgery, Jena University Hospital, Germany. Eligibility required available neuroimaging and in-hospital outcome data. Patients with a documented or subsequently confirmed tumor, vascular lesion, or traumatic injury were excluded (Supplementary Fig. S1).

The cohort comprised only surgically treated patients and therefore does not represent the broader population of patients with cerebellar stroke. Surgical indications included neurological deterioration, mass effect, fourth-ventricle or brainstem compression, and obstructive hydrocephalus, as appropriate for the underlying pathology.

Cases of hemorrhagic transformation of cerebellar infarction (small petechial hemorrhage within a predominantly ischemic lesion, n = 3) were classified within the ischemic stroke group because the predominant radiological finding was infarction rather than primary hemorrhage. This classification was based on the initial CT findings and corresponding neuroradiological reports, which characterized these lesions as cerebellar infarctions with secondary hemorrhagic transformation (Von Kummer et al. 2015). The primary endpoint was all-cause mortality during the index hospitalization. The inclusion of hemorrhagic and ischemic cerebellar lesions, including hemorrhagic transformation, was intentional, as the study focused on patients requiring posterior fossa decompression for space-occupying cerebellar pathology rather than on a specific stroke etiology. This approach allowed the exploratory assessment of anatomical and volumetric characteristics associated with in-hospital mortality across different stroke subtypes sharing a common surgical indication.

Fourth-ventricle compression was categorized as incomplete when the ventricular lumen remained at least partially identifiable and complete when it was no longer radiographically discernible because of mass effect (Supplementary material)

For clarification and standardization of the procedure, a detailed description of the surgical technique is provided in the supplementary information.

## 3 Volumetric and index measurements

The following measurements were obtained from preoperative and postoperative CT acquisitions using Brainlab software (SmartBrush Tool, Brainlab AG, Munich, Germany): lesion volume (VL; infarct or hematoma volume), cerebellar volume (VC; total cerebellar volume including the lesion and edema), and posterior fossa volume (VPF; intracranial posterior fossa space). These measurements are represented in the Supplementary Fig. S2. Fourth-ventricle compression was evaluated in the preoperative CT scan and classified as incomplete or complete. The maximal axial and sagittal dimensions of the achieved decompression were measured on postoperative CT and were considered measures of procedural extent rather than preoperative anatomical predictors. To ensure consistency in image interpretation and measurement technique, all measurements were performed by a single assessor (LK). The assessor was blinded to the in-hospital outcome to reduce the potential for observer bias.

### 3.1 Statistical analysis

All analyses were performed using R (version 4.2), Python (latest version at time of analysis), and IBM SPSS Statistics (version 29.0.2.0 (20) IBM Corporation, 2023). Spearman's rank correlation was used for the matrix correlation to assess linear relationships among variables. Group comparisons were conducted with Fisher's exact tests for categorical variables and Mann-Whitney U tests for continuous variables. Statistical significance was set at p < 0.05. Given the small number of mortality events relative to the candidate variables, LASSO (Least Absolute Shrinkage and Selection Operator) logistic regression was used as an exploratory, hypothesis-generating method for signal screening and variable prioritization. By penalizing the absolute magnitude of regression coefficients, LASSO simultaneously shrinks weak or redundant effects towards zero and retains a more parsimonious subset of variables, thereby reducing the influence of multicollinearity and facilitating interpretation in this small cohort. The model evaluated a comprehensive set of clinical and structural variables, including type of stroke: hemorrhage vs. infarction, location (unilateral vs. bilateral), 4th ventricle compression, surgical approach (unilateral vs. bilateral decompression), pre-surgical GCS, posterior fossa and cerebellar volume. To ensure clinical relevance and ease of interpretation, standardized coefficients are presented in original units to report Odds Ratios (OR) per 1-unit increase for continuous variables or per category presence for binary indicators. However, in this small cohort, retention should not be interpreted as evidence of an independent association, a stable effect-size estimate, or validated predictive performance. Accordingly, the resulting coefficients and odds ratios were considered descriptive indicators of the direction and relative prominence of candidate. To assess the stability of variable selection and reduce the risk of overinterpreting a single penalized model, we additionally performed bootstrap stability selection using 1000 resamples. The LASSO procedure was refitted in each resample, and the proportion of resamples in which each variable was retained by the penalty was recorded as its selection frequency.

## 4 Results

### 4.1 Demographics and clinical characteristics

The study cohort comprised 23 patients undergoing surgical intervention for cerebellar insult. As detailed in Table 1, the median age of the population was 72 years, with a sex distribution of 15 males versus 8 females. Patients who survived exhibited significantly longer overall lengths of stay (median 25 days vs. 5 days; p = 0.009), this comparison is descriptive because death itself truncates hospitalization. Exploratory inspection of the Spearman correlation matrix suggested correlation patterns between mortality and type of stroke, lesion location, fourth-ventricle compression, and the extent of posterior fossa decompression; however, because no p values were calculated, these findings should be interpreted descriptively and not as evidence of statistical association (Supplementary Table 1).

**Table 1:** Baseline clinical, radiological, and surgical characteristics stratified by in-hospital survival status (survived n = 17 vs dead n = 6). Categorical variables are reported as n; continuous variables are reported as median (IQR). P-values compare survivors vs non-survivors (two-sided; Fisher's exact test for categorical variables and Mann–Whitney *U* test for continuous variables, given the small sample size). Abbreviations: preGCS: preoperative Glasgow Coma Scale; EVD: external ventricular drain; LOS, length of hospital stay (days).

Variable	Survived (n = 17)	Dead (n = 6)	p-value
Clinical Data			
**Age**	65.36 (60.56-76.25)	75.33 (57.78-80.93)	0.562
**Sex**			1.000
**male**	11 (65%)	4 (67%)	
**female**	6 (35%)	2 (33%)	
**Type of stroke**			0.014*
**Hemorrhage**	6 (35%)	6 (100%)	
**Ischemia**	11 (65%)	0	
**Location**			0.059
**rigth**	5 (29.5%)	0	
**left**	6 (35%)	0	
**midline**	1 (5%)	1 (17%)	
**crossing midline**	5 (29.5%)	5 (83%)	
**preGCS**	8.00 (3.00-10.00)	5.50 (3.00-8.00)	0.288
**supratentorial findings**			1.000
**yes**	4 (23%)	1 (17%)	
**no**	13 (77%)	5 (83%)	
**Hydrocephalus**			0.539
**yes**	14 (82%)	6 (100%)	
**no**	3 (18%)	0	
**Shunt**			0.369
**yes**	7 (41%)	1 (17%)	
**no**	10 (59%)	5 (83%)	
**4th Ventricle Compression**			0.052
**incomplete**	12 (70.5%)	1 (17%)	
**complete**	5 (29.5%)	5 (83%)	
**EVD**			1.000
**yes**	16 (95%)	6 (100%)	
**no**	1 (5%)	0	
**Lesion Resection**			1.000
**yes**	15 (88%)	6 (100%)	
**no**	2 (12%)	0	
**Bilateral decompression**			0.052
**bilateral**	5 (29.5%)	5 (83%)	
**unilateral**	12 (71.5%)	1 (17%)	
**Reoperation**			0.660
**yes**	8	2 (33%)	
**no**	9	4 (67%)	
**Decompr, Extension**			1.000
**yes**	3 (18%)	1 (17%)	
**no**	14 (82%)	5 (83%)	
**CSF Leakage**			1.000
**yes**	5 (29%)	1 (17%)	
**no**	12 (71%)	5 (83%)	
**Time to surgery (d)**	0.00 (0.00-1.00)	0.00 (0.00-0.00)	0.050
**LOS (d)**	25.00 (20.00–29.00)	5.00 (2.50–10.50)	0.009*
**Mesurement Data**			
**Axial (cm)**	4.64 (3.70-6.59)	5.65 (4.17-6.07)	0.889
**Sagittal (cm)**	4.20 (3.40-4.79)	3.80 (3.60-4.00)	0.380
**Lesion Volume (cm3)**	38.60 (27.20-62.80)	33.15 (19.85-33.92)	0.227
**Posterior Fossa Volume (cm3)**	197.70 (188.30-213.90)	198.55 (189.15-209.68)	0.812
**Cerebellar Volume (cm3)**	156.20 (139.80–170.20)	157.35 (140.62-177.22)	0.810

Clinically the type of cerebellar stroke was associated with in-hospital mortality in this cohort (p = 0.014); while survivors were a mix of hemorrhage and infarction, all deaths occurred exclusively in patients with hemorrhage, as visualized in Fig. 1A. In-hospital mortality was 50.0% (6/12) among patients with hemorrhage versus 0% (0/11) among patients with ischemic stroke, a risk difference of 50.0 percentage points (95% CI 14.3–74.6, Newcombe method). Furthermore, patients who died presented with a lower median pre-surgical GCS (5.5 vs. 8.0), although it did not reach independent significance in the univariate frequency analysis (p = 0.288).

**Fig. 1:**
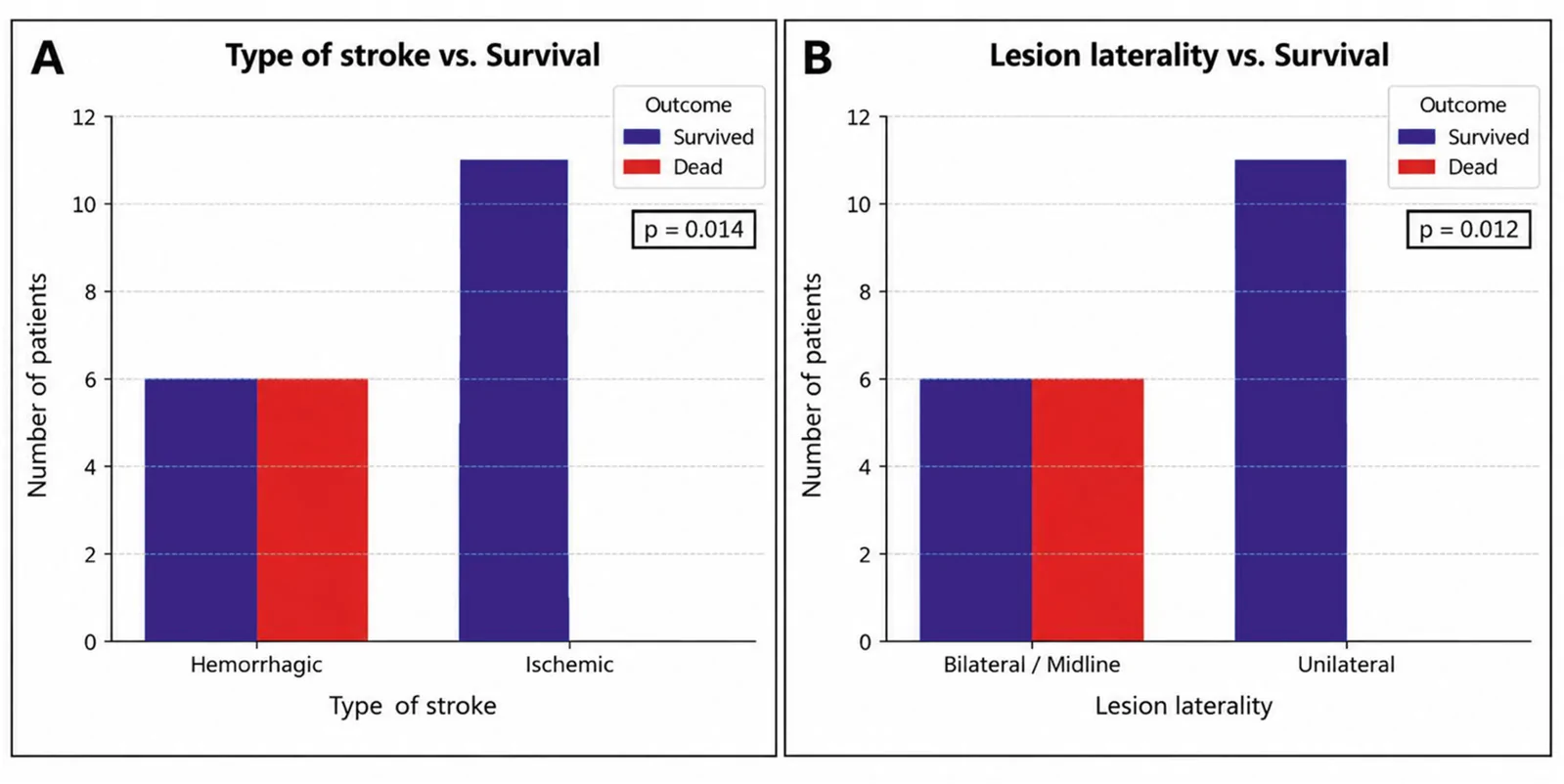
Distribution of survival status according to type of stroke (A) and lesion location (B). The difference between groups was statistically significant for diagnostic (p = 0.014, Fisher's exact test) and for lesion location (p = 0.012, Fisher's exact test).

### 4.2 Anatomical and volumetric paraclinical indicators

Anatomical, radiological, surgical, and quantitative measurement variables were compared between the 17 survivors and 6 non-survivors. Lesion distribution showed the clearest anatomical pattern in this exploratory cohort. All patients with strictly unilateral lesions survived: five patients had right-sided lesions and six had left-sided lesions, with no deaths in either subgroup. In contrast, one of the two patients with a midline lesion and five of the ten patients with lesions crossing the midline died. The overall comparison across the four original location categories was suggestive but did not reach the prespecified significance threshold (p = 0.059). When location was dichotomised on clinical grounds as unilateral versus bilateral or midline involvement, the latter category was associated with in-hospital mortality (p = 0.012; Fig. 1B). This pattern suggests that the spatial extent and central involvement of the lesion may be more informative than laterality alone, although the small numbers within each category warrant cautious interpretation.

Fourth-ventricle compression demonstrated a similar descriptive pattern. Complete compression was observed in five of six non-survivors and five of seventeen survivors, whereas incomplete compression was present in one of six non-survivors and twelve of seventeen survivors (p = 0.052). Thus, complete compression was substantially more frequent among patients who died, but the comparison remained just above the conventional threshold for statistical significance.

Bilateral decompression had an identical distribution—five of six non-survivors and five of seventeen survivors underwent bilateral decompression, compared with unilateral decompression in one of six and twelve of seventeen, respectively (p = 0.052). Because the extent of decompression was determined after clinical and radiological assessment, this finding is more plausibly interpreted as a marker of disease extent and treatment selection than as evidence of an adverse effect of bilateral surgery.

Quantitative volumetric and linear measurements showed no clear separation between outcome groups. Median lesion volume was lower in non-survivors than in survivors (33.15 vs 38.60 cm^3^), but this difference was not statistically significant (p = 0.227). Posterior fossa volume was nearly identical between groups (198.55 vs 197.70 cm^3^; p = 0.812), as was cerebellar volume (157.35 vs 156.20 cm^3^; p = 0.810). The median postoperative axial decompression dimension was 5.65 cm in non-survivors and 4.64 cm in survivors (p = 0.889), while the corresponding sagittal dimensions were 3.80 and 4.20 cm (p = 0.380). Collectively, these findings provide no evidence that absolute lesion volume, posterior fossa volume, cerebellar volume, or postoperative decompression dimensions differed according to survival status in this cohort. Overall, lesion distribution and fourth-ventricle compression emerged as the principal anatomical signals, warranting further evaluation as hypothesis-generating markers rather than established prognostic factors.

### 4.3 LASSO logistic regression

The final LASSO model identified the most parsimonious set of factors associated with death, excluding non-contributory variables like cerebellar volume (Table 2). As illustrated in the LASSO Fig. 2, the model retained hemorrhagic stroke (OR 4.40), bilateral lesion involvement (OR 3.64), and complete fourth-ventricle compression (OR 1.35), corresponding to higher estimated odds of mortality in this exploratory cohort. Notably, preGCS (OR 1.00), and cerebellar volume were non selected as variables related to death in this model. Posterior fossa volume remained in the model as a weak protective signal (OR 0.998), such that each 1 cm^3^ increase in PFV was associated with an approximately 0.2% lower odds of death. These are descriptive penalized model coefficients, not independent predictors or reliable effect-size estimates. As an additional safeguard against overstating precision, we performed bootstrap stability selection using 1000 resamples and recorded the proportion of resamples in which each variable was retained by the LASSO penalty. Hemorrhagic diagnosis and bilateral or midline lesion location were retained in the large majority of resamples, supporting the relative stability of these two signals, whereas posterior fossa volume and pre-surgical GCS were selected less consistently. These selection frequencies are presented in Supplementary Fig. S3 to convey the stability of the exploratory findings without implying a degree of inferential precision, such as that conveyed by confidence intervals, that the sample size cannot support.

**Table 2:** Results of the exploratory LASSO logistic regression model evaluating candidate variables associated with in-hospital death. Standardized coefficients and non-standardized coefficients from the penalized logistic regression are presented together with the corresponding odds ratios (OR) per original unit of measurement. Variables with coefficients shrunk to zero by the LASSO penalty were effectively excluded from the final model, indicating no detectable independent association within this dataset. Abbreviations: St. Coefficient: standardized regression coefficient; β: non-standardized regression coefficient; OR: odds ratio; LASSO: least absolute shrinkage and selection operator; cm^3^, cubic centimeters.

Variable	St. Coeficient	β (per original unit)	OR (per unit)	Clinical Interpretation
**Hemorrhage**	0.740	1.482	4.400	Hemorrhage increases odds of death by ∼3,4x.
**Bilateral Lesion**	0.645	1.293	3.640	Bilateral location increases odds of death by ∼2,6x.
**Complete Compression**	0.147	0.297	1.350	Complete 4th ventricle compression increases odds by ∼35%.
**Bilateral Decompression**	0.000	0.000	1.000	No independent effect (excluded by model).
**Cerebellar Volume (cm3)**	0.000	0.000	1.000	No independent effect (excluded by model).
**Posterior fossa volume (cm3)**	−0.037	−0.002	0.998	No independent effect (near to one).
**preGCS**	0.000	0.000	1.000	No independent effect (excluded by model).

**Fig. 2:**
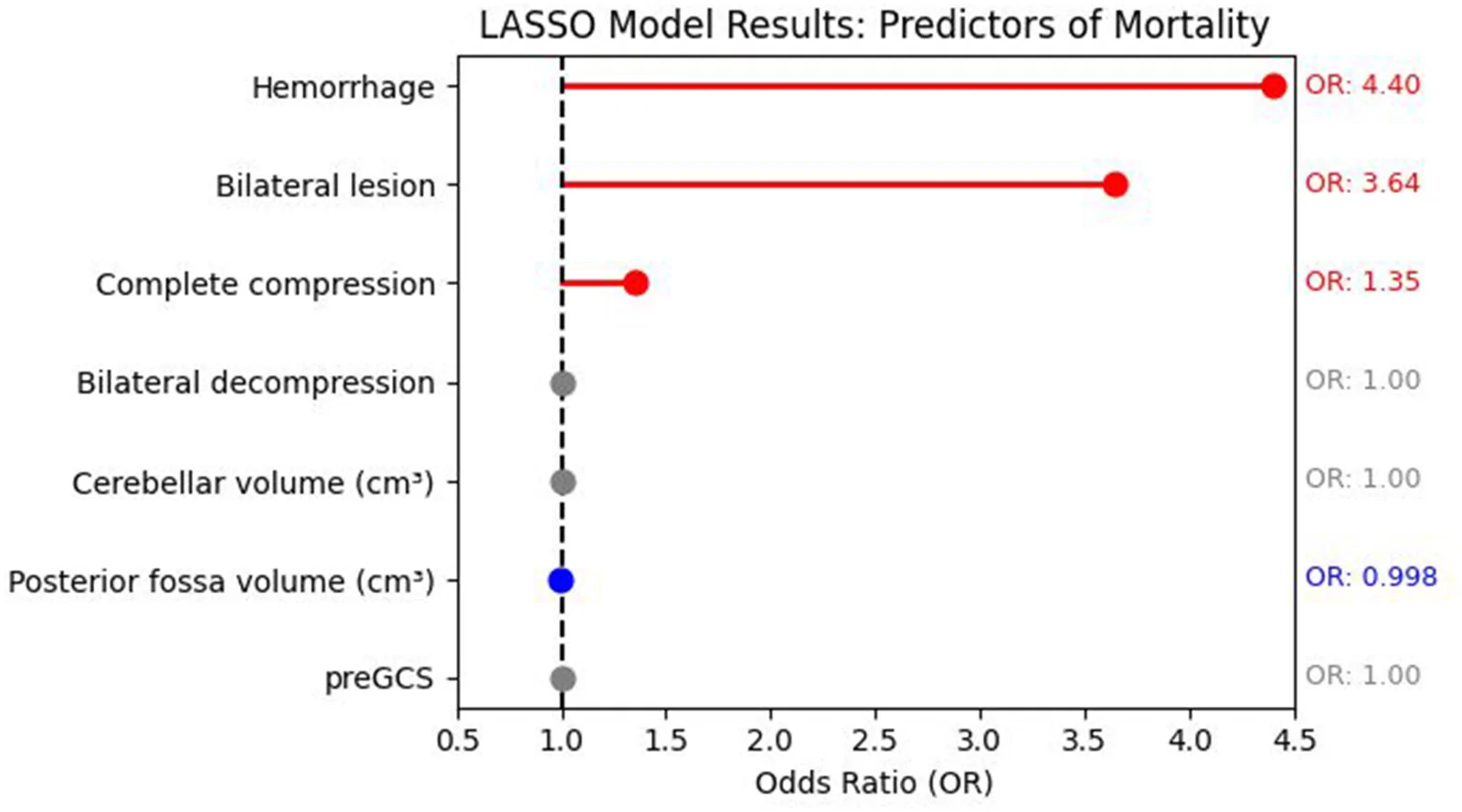
Results of the exploratory LASSO logistic regression model evaluating candidate variables associated with in-hospital death. Odds ratios (OR) are shown for each variable retained in the penalized model, with the vertical dashed line indicating the null value (OR = 1). Variables with coefficients shrunk toward zero by the LASSO penalty (e.g., bilateral decompression, cerebellar volume, preoperative Glasgow Coma Scale) showed no detectable independent association in this dataset. Abbreviations: OR, odds ratio; LASSO, least absolute shrinkage and selection operator; preGCS, preoperative Glasgow Coma Scale.

## 5 Discussion

In this exploratory, single-center study, we assessed associations between clinical, anatomical, and volumetric factors and acute in-hospital mortality among adults undergoing surgical treatment for cerebellar insult. Using descriptive statistics, correlation analysis, dimensionality reduction, and penalized regression modelling, we sought to identify candidate signals associated with early postoperative mortality in this small cohort. Our results indicate that the type of stroke, lesion topology —rather than absolute volumetric burden—are the primary drivers of in-hospital mortality in this surgically treated cohort.

### 5.1 The role of etiology and neurological status

The type of cerebellar stroke emerged as a major determinant of outcome in our cohort. All acute in-hospital deaths occurred in patients with cerebellar hemorrhage, whereas survivors represented a mixed population of hemorrhagic and ischemic insults. This finding is consistent with multiple prior studies demonstrating higher early mortality in hemorrhagic compared with ischemic cerebellar lesions, even in surgically managed patients (Dammann et al., 2011; Lee et al., 2020). Hemorrhagic pathology is associated with rapid mass effect, abrupt posterior fossa crowding, and early brainstem compression, factors that may not be fully reversible despite timely decompression.

Preoperative neurological status, reflected by the Glasgow Coma Scale (GCS), showed a clear trend toward lower values in non-survivors. However, this variable did not independently discriminate mortality in univariable analyses nor emerge as a dominant predictor in multivariable modeling. This observation aligns with previous reports suggesting that while GCS reflects disease severity at presentation, its prognostic value may be overshadowed by anatomical constraints within the posterior fossa once critical compression has occurred (Dahdaleh et al., 2012; Tsitsopoulos et al., 2011). In this context, neurological status appears to function as a secondary marker of underlying structural compromise rather than an independent driver of outcome.

### 5.2 Lesion location and ventricular compression as key predictors

Anatomical lesion characteristics proved to be central to mortality risk. Lesions involving the midline or crossing into bilateral cerebellar territories were associated with higher mortality rates, whereas unilateral lesions were predominantly observed among survivors. Midline involvement likely reflects greater proximity to the brainstem and fourth ventricle, increasing the risk of rapid neurological deterioration (Picelli et al., 2017; Tsitsopoulos et al., 2011). Fourth-ventricle compression showed a descriptive correlation with mortality, a borderline univariable association, and was retained in the exploratory penalized model. However, this should be interpreted cautiously given the small sample size.

Recent evidence from a larger cohort of 194 patients with spontaneous cerebellar hemorrhage demonstrated that neurological status, age, and hematoma volume were independently associated with six-month mortality, whereas fourth-ventricle and brainstem compression showed associations only in univariable analyses (Sida et al., 2026). These findings support the relevance of neurological and radiological markers in cerebellar hemorrhage, although differences in stroke etiology, treatment selection, and outcome assessment limit direct comparison with our surgically treated cohort and its in-hospital mortality endpoint.

These findings reinforce earlier observations that ventricular obstruction and brainstem compression are decisive prognostic features in cerebellar pathology (Dammann et al., 2011; Luney et al., 2016). From a pathophysiological perspective, fourth-ventricle compression serves as a surrogate for critical posterior fossa crowding, impaired CSF circulation, and imminent secondary injury—mechanisms directly linked to early mortality (Dahdaleh et al., 2012; Wijdicks et al., 2014).

### 5.3 Volumetric measurements: prognostic value for early mortality

A secondary objective of this study was to assess whether quantitative volumetric measurements of the cerebellum, lesion, and posterior fossa could improve mortality prediction. Contrary to expectations, none of the evaluated volumetric parameters—including lesion volume, cerebellar volume, posterior fossa volume, or linear diameters—demonstrated a significant or consistent association with in-hospital early mortality, but the small sample and limited variation in outcome substantially reduce power. In general, these findings contrast with recent work suggesting that volumetry may aid in determining surgical indications or predicting functional outcome after cerebellar infarction (Kapapa et al., 2024; Wang et al., 2022). However, it is important to note that most of these studies focused on decision-making thresholds or long-term outcomes, rather than early mortality in patients who had already undergone surgery. In our cohort, surgical decompression may have mitigated the prognostic impact of absolute volume, rendering static measurements less informative than dynamic indicators of posterior fossa compromise, such as ventricular compression.

### 5.4 Insights of multivariate structure and predictive modeling

LASSO logistic regression further refined this pattern by identifying a parsimonious set of independent predictors in this cohort. Hemorrhagic etiology, bilateral or midline lesion involvement, bilateral decompression and complete fourth-ventricle compression emerged as the most influential contributors to mortality risk, while volumetric variables were consistently penalized and excluded from the final model. Interestingly, bilateral decompression was associated with an approximately 22% higher risk of death. This finding should not be interpreted as a detrimental effect of the surgical technique itself, but rather as a reflection of disease severity and surgical selection bias. Thus, bilateral decompression in this context likely serves as a surrogate marker of extreme anatomical and physiological compromise (Hernandez-Duran et al., 2025; Luney et al., 2016). Importantly, it appeared associated with higher mortality, likely reflecting severity-driven surgical selection rather than a harmful effect of the procedure itself.

In a multicenter cohort of 531 patients with cerebellar infarction demonstrated that the association between surgical treatment and functional outcome varied according to infarct volume, although mortality did not differ significantly between treatment groups after propensity-score matching (Won et al., 2024). Similarly, a recent multicenter study involving 88 surgically treated patients with cerebellar infarction found that larger postoperative decompression dimensions were associated with favorable functional outcomes but not with a statistically significant difference in three-month mortality (Hernández-Durán et al., 2026). These observations highlight the importance of distinguishing functional recovery, surgical selection, and mortality as separate endpoints when interpreting volumetric and anatomical measurements.

All together, these findings add importance of recent efforts to develop clinical grading systems for cerebellar stroke (Hernández-Durán et al., 2026; Won et al., 2024). Our findings prioritize anatomical and clinical markers over absolute size metrics. These data cannot determine whether surgery should be performed or withheld, because no nonsurgical comparator group was included. The present observations may inform hypotheses for future evaluation of lesion distribution and posterior fossa compromise, not clinical decision thresholds. Simple and readily available imaging markers—particularly fourth-ventricle compression and lesion location—may therefore provide more clinically actionable prognostic information than complex volumetric analyses in the acute setting.

Future studies should aim to validate these findings in larger, multicenter cohorts and explore the role of dynamic imaging markers, such as edema progression or changes in ventricular patency over time. Integrating volumetry with measures of posterior fossa compliance and CSF dynamics may further refine risk stratification and surgical timing strategies.

### 5.5 Clinical implications

Although exploratory, our findings highlight the importance of considering type of stroke and lesion distribution when assessing patients undergoing posterior fossa decompression. In our cohort, hemorrhagic lesions and midline or bilateral cerebellar involvement were associated with higher in-hospital mortality, while fourth-ventricle compression showed a possible association. These readily identifiable radiological features may complement neurological assessment and support clinical vigilance in patients at risk of deterioration. Importantly, the absence of significant associations with volumetric measurements does not diminish their potential clinical value. Given the small cohort, these observations should inform clinical awareness rather than guide surgical decisions or establish prognostic thresholds.

### 5.6 Limitations

This retrospective single-center analysis included only 23 operated patients and six deaths, severely limiting statistical power and making penalized coefficients sensitive to case selection and model tuning. The inclusion of both hemorrhagic and ischemic cerebellar lesions introduces etiological heterogeneity, which may have influenced the observed associations with mortality. Although these conditions differ in their initiating pathophysiological mechanisms, they share important consequences related to space-occupying lesions within the confined posterior fossa. Selection for surgery, confounding by indication for bilateral decompression, and residual confounding by unmeasured disease severity prevent causal inference or extrapolation to nonsurgical patients. Imaging segmentation and classification may introduce measurement bias; reliability could not be assessed from the currently documented methods. Static volumes and postoperative decompression dimensions do not capture the evolving preoperative mass effect. Multiple exploratory comparisons were not adjusted for multiplicity. No validated prediction model or external validation is available. Non-significant volumetric results should not be interpreted as evidence of no association.

## 6 Conclusion

In this exploratory cohort of 23 surgically treated adults, all six in-hospital deaths occurred among patients with hemorrhagic stroke, and bilateral/midline-crossing lesion involvement was more frequent among non-survivors. Complete fourth-ventricle compression was descriptively more frequent in non-survivors but did not meet the prespecified significance threshold. No statistically significant association with absolute volumetric measurements was detected; this finding may reflect limited statistical power. Exploratory LASSO coefficients should not be interpreted as independent predictors or used to guide surgical selection. Larger, prospectively characterized cohorts are required to assess the reproducibility and clinical relevance of these observations.

## Ethics

This study was performed in accordance with institutional guidelines for retrospective research and approved by the Jena University Hospital institutional review board (2025-3910-BO-D). The requirement for informed consent was waived due to the use of anonymized data.

## Author contributions

This study was conceptualized and designed by SACM and NA. Data was gathered by SACM and NA. LK performed the CT measurements using Brainlab software. Statistical analysis was planned and performed by SACM, RB and DS. Interpretation of results was performed by all authors. The manuscript was written by SACM. All authors read and approved the final manuscript.

## Further contributions

AI-based language assistance (Microsoft Copilot, powered by GPT-4/Microsoft 365) was used to improve grammar and clarity. All content was critically reviewed and verified by the authors.

## Data availability

The dataset is not publicly available due to conditions of the ethics approval. Data on a cohort level may be made available by the corresponding author upon reasonable request.

## Funding

This research did not receive any specific grant from funding agencies in the public, commercial, or not-for-profit sectors.

## Declaration of competing interest

The authors declare that they have no known competing financial interests or personal relationships that could have appeared to influence the work reported in this paper.
