# Supplementary material for: Predictors of in-hospital mortality after posterior fossa decompression for cerebellar stroke: an exploratory cohort study
Source: Brain Spine. 2026 Nov 1;6:107721. doi: 10.1016/j.bas.2026.107721 (PMC13755594; doi:10.1016/j.bas.2026.107721)
Supplement: Supplementary file 1 [file mmc1.docx]

Surgical technique:

All surgical procedures were performed under general anesthesia, with the patient first in supine position for the placement of an EVD (n=22) and then in prone position. The cranium was secured in a three-pin Mayfield head holder with head flexion. For the bilateral decompression, a midline suboccipital approach was utilized, which was then followed by a craniectomy of the occipital bone on both sides, extending caudally to the foramen magnum. For the unilateral decompression, a paramedian suboccipital approach was employed, involving a unilateral craniectomy that extended medially to the midline and caudally to the foramen magnum. In all patients, following dural opening, the arachnoid membrane of the cisterna magna was opened to allow cerebrospinal fluid drainage and facilitate posterior fossa decompression. In patients with cerebellar hemorrhage, surgical evacuation of the hematoma was undertaken. In patients with cerebellar infarction, necrotic tissue was debrided to provide additional decompression. The dura mater was left open, without primary closure, only with on-lay TachoSil. The resected bone was not replaced. The wound was closed in layers, involving the approximation of muscles and fascia, followed by subcutaneous sutures and skin closure.

**Supplementary figures**

**Suppl. Figure S1**

Supplementary Figure S1. Patient selection flow diagram. Patients were identified through cerebellar stroke diagnosis codes (using ICD-10) and posterior fossa decompression procedures, cross-referenced with the institutional neurosurgical registry. Of 26 potentially eligible patients, three were excluded following medical record review (two with traumatic injuries and one with an underlying vascular lesion). The final study cohort comprised 23 patients undergoing posterior fossa decompression for cerebellar stroke.

**Suppl. Figure S2**

Supplementary Figure S2. Representative axial, sagittal, and coronal CT images showing volumetric segmentation of the posterior fossa (blue), cerebellum (yellow), and cerebellar lesion (red) (A cerebellar infaction and B cerebellar hemorrhage). Additional axial and sagittal images illustrate (C) incomplete (partially visible lumen) and (D) complete (no discernible lumen) fourth-ventricle compression due to mass effect.

**Suppl. Figure S3**

Supplementary Figure S3. Stability of LASSO variable selection. Bootstrap selection frequencies of candidate variables in LASSO logistic regression across 1,000 resamples. The dashed vertical line indicates a 50% selection-frequency threshold. Colors distinguish variables retained in the original model with positive or negative coefficients from those excluded. Higher frequencies indicate greater selection stability, not statistical significance or independent predictive value.

**Supplementary tables**

**Suppl. Table S1**

|  | **deaths** |
| --- | --- |
| **deaths** | 1 |
| **Age** | 0.13434332265597 |
| **Sex** | -0.0180775381515547 |
| **Axial (cm)** | -0.0373360408463897 |
| **Sagittal (cm)** | -0.194629288080143 |
| **Lesion Volume (cm3)** | -0.268686645311939 |
| **Posterior Fossa Volume (cm3)** | -0.0746351792533165 |
| **Cerebellar Volume (cm3)** | 0.104489250954643 |
| **Diagnosis** | **-0.568796458994521** |
| **Location** | **0.521909144707537** |
| **preGCS** | -0.234515689879414 |
| **supratentorial findings** | 0.0730595006266008 |
| **Hydrocephalus** | -0.230089496654211 |
| **Shunt** | 0.225969226894433 |
| **4th Ventricle Compression** | 0.477629263575664 |
| **EVD** | -0.126660099276225 |
| **Lesion Resection** | -0.183339699405642 |
| **Bilateral decompression** | -0.477629263575664 |
| **Reoperation** | 0.121578358001078 |
| **Decompr. Extension** | 0.0113577712606064 |
| **CSF Leakage** | 0.127450980392157 |
| **Time to surgery (d)** | -0.426357549352278 |
|  |  |
| Supplementary Table 1. Spearman correlation matrix of clinical and imaging variables. Values represent Spearman’s rank correlation coefficients (ρ). Death (0 = survived, 1 = died) is shown as the first row/column to facilitate identification of associations with in-hospital mortality. Spearman correlation was chosen to account for non-normally distributed variables and ordinal/coded clinical data. Positive values indicate direct associations, and negative values indicate inverse associations. | |
